# Effects of omega-3 supplementation on components of the endocannabinoid system and metabolic and inflammatory responses in adipose and liver of peripartum dairy cows

**DOI:** 10.1186/s40104-022-00761-9

**Published:** 2022-10-02

**Authors:** Gitit Kra, Jayasimha Rayalu Daddam, Uzi Moallem, Hadar Kamer, Radka Kočvarová, Alina Nemirovski, G. Andres Contreras, Joseph Tam, Maya Zachut

**Affiliations:** 1 Department of Ruminant Science, Institute of Animal Sciences, ARO Volcani Institute, Rishon LeZion, Israel; 2 Department of Animal Science, The Robert H. Smith Faculty of Agriculture, Food and Environment, The Hebrew University of Jerusalem, Rehovot, Israel; 3 Obesity and Metabolism Laboratory, The Institute for Drug Research, School of Pharmacy, Faculty of Medicine, The Hebrew University of Jerusalem, Jerusalem, Israel; 4 Department of Large Animal Clinical Sciences, College of Veterinary Medicine, Michigan State University, East Lansing, Michigan USA

**Keywords:** Adipose, Dairy cows, Endocannabinoid system, Liver, Omega-3

## Abstract

**Background:**

Dietary supplementation of omega-3 fatty acids can reduce the activation of the endocannabinoid system (ECS) by decreasing the availability of arachidonic acid, thus lowering endocannabinoids (eCBs) levels. The ECS is a modulator of energy metabolism, stress response and inflammation in mammals, yet there is little information on the roles of the ECS in transition dairy cows. During the periparturient period, the adipose tissue and liver are the main metabolic organs that participate in the adaptations of dairy cows to onset of lactation; however, exceeded adipose tissue lipolysis and accumulation of lipids in the liver have adverse effects on cows’ physiology. Here we aimed to examine whether omega-3 supplementation during the transition period will modulate ECS activation and affect metabolic and inflammatory indices in postpartum dairy cows, by supplementing twenty-eight transition Holstein dairy cows with either saturated fat (CTL) or encapsulated flaxseed oil (FLX). Components of the ECS, metabolic and inflammatory markers were measured in blood, liver, and subcutaneous adipose tissue.

**Results:**

FLX supplementation reduced feed intake by 8.1% (*P* < 0.01) and reduced plasma levels of arachidonic acid (by 44.2%; *P* = 0.02) and anandamide (by 49.7%; *P* = 0.03) postpartum compared to CTL. The mRNA transcription levels of the cannabinoid receptor 1 (*CNR1*/CB1) tended to be lower (2.5 folds) in white blood cells of FLX than in CTL (*P* = 0.10), and protein abundance of ECS enzyme monoacylglycerol lipase was higher in peripheral blood mononuclear cells of FLX than in CTL (*P* = 0.04). In adipose tissue, palmitoylethanolamide levels were lower in FLX than in CTL (by 61.5%; *P* = 0.02), relative mRNA transcription of lipogenic genes were higher, and the protein abundance of cannabinoid receptor 2 (*P* = 0.08) and monoacylglycerol lipase (*P* = 0.10) tended to be higher in FLX compared to CTL. Hepatic 2-arachidonoylglycerol tended to be higher (by 73.1%; *P* = 0.07), and interlukin-6 mRNA transcription level was 1.5 folds lower in liver of FLX than in CTL (*P* = 0.03).

**Conclusions:**

Nutritional supplementation of omega-3 fatty acids seems to partly modulate ECS activation, which could be related to lower feed intake. The altered ECS components in blood, adipose tissue and liver are associated with moderate modulations in lipid metabolism in the adipose and inflammation in liver of peripartum dairy cows.

**Supplementary Information:**

The online version contains supplementary material available at 10.1186/s40104-022-00761-9.

## Background

The transition period from late pregnancy to calving and the onset of lactation is a crucial time for dairy cows, and is characterized by several changes in endocrine, metabolic, and immune functions. Within 4 days of lactation, the mammary gland uptake for glucose, amino acids and fatty acids are several folds more than those of the fetus and utero-placenta before the term of pregnancy [1]; however, the cow’s feed intake is limited during this period, which leads to a negative energy balance. When energy supply is limited, fatty acids from the adipose tissue (AT) are used as a source of energy and this initiates lipolysis in the AT [2]. Metabolic adaptations such as lipid mobilization are accompanied by alterations in inflammatory and immune responses and functions [2]. During the peripartum period, cows also experience systemic subacute inflammation, which is accompanied by a mild increase in pro-inflammatory mediators [3]. In most cows, negative energy balance and lipolysis decrease, and AT inflammation resolves as lactation progresses. However, if lipolysis dysregulation occurs and lipolysis rate does not decrease, AT inflammation will become chronic and lead to high disease susceptibility, poor lactation performance, reproductive failure, and may lead to increased risk for culling [3].

Numerous studies have shown that dietary omega-3 (n-3) fatty acids have positive effects on reproductive and physiological properties in dairy cows [4–8]. In addition, changing omega-6 (n-6) to n-3 ratio in the diet is a well-known strategy for affecting the endocannabinoid system (ECS), and the possible consequences of this change in ECS activity on peripartum dairy cows is yet to be explored [9, 10]. In dairy cows, the AT and liver have a major role in energy metabolism, and the ECS may play a crucial role in activation of lipogenesis and adipogenesis as well as inhibition of lipolytic activity [11]. Previously, the presence of key elements of the ECS in subcutaneous AT of dairy cows was established, and elevated levels of endocannabinoids (eCBs) were measured in postpartum (PP) AT [9]. The ECS may also be related to inflammation in AT of dairy cows, as the presence of many inflammatory mediators were found to be higher in AT of cows with high lipolysis PP, coupled with higher expression of the cannabinoid-2 (CB2) receptor, and the enzymes required for the synthesis and degradation of one of the main eCB anandamide (AEA) [12]. The ECS is also an integral regulator of the stress response; across different stress paradigms, stress evokes bidirectional changes in AEA and 2-arachidonoylglycerol (2-AG), reducing AEA levels and increasing 2-AG levels [13]. The decline in AEA appears to activate the hypothalamic–pituitary–adrenal (HPA) axis, while the increased 2-AG signaling contributes to termination and adaptation of the HPA axis [13]). As PP cows are exposed to various stressors, the possible effects of modulating ECS activity on their HPA axis stress response is of interest.

As mentioned above, dietary supplementation of n-3 fatty acids is a strategy to decrease the activation of the ECS by lowering the availability of arachidonic acid (AA; C20:4n-6), a precursor of eCBs synthesis and eCBs levels in tissues [9, 10]. The eCBs AEA and 2-AG are synthesized from AA and are hydrolyzed to AA and ethanolamine and glycerol by the enzymes fatty acid amide hydrolase (FAAH) and monoacylglycerol lipase (MGLL), respectively [14]. Flaxseed oil is one of the richest sources of the n-3 fatty acid alpha-linolenic acid (C18:3n-3) [15]. At this time, it is not known whether reducing the ECS activity by n-3 supplemental diet could be beneficial to peripartum dairy cows; on one hand, it might promote AT lipolysis and lower intake, but on the other hand it might decrease inflammation. We hypothesized that supplementing peripartum cows with n-3 fatty acids will lower the systemic availability of AA and reduce ECS activation [16] in blood, liver and AT and influence the metabolic, stress-related and inflammatory responses of PP dairy cows.

## Methods

### Animals and experimental procedures

The experimental protocol for the study was approved by the Volcani Center Animal Care Committee (approval number IL 797/18), and was performed in accordance with the relevant guidelines and regulations. The experiment was conducted at the Volcani Center experimental dairy farm in Rishon Lezion, Israel. The experimental design was previously reported in detail [17]. Briefly, the experiment was conducted with twenty-eight multiparous (mean parity 3.8 ± 1.4; mean ± SD) high-yielding Israeli-Holstein dairy cows at 255 ± 5 d of pregnancy, during the winter season. The cows were group-housed in a shaded loose pen that was equipped with a real-time electronic individual feeding system. The cows were stratified according to milk yields during the first 60 d of previous lactation, body weight (BW) at drying off and parity. The cows were examined by a veterinarian 5 to 10 d postpartum (PP), according to the routine management in Israel. The dietary treatments started at 257 d of pregnancy and continued until 60 d in lactation as follows: (i) CTL (*n* = 14) — fed a basal diet, supplemented with encapsulated saturated fat at 240 and 560 g/d per cow prepartum and PP, respectively; (ii) FLX (*n* = 14) — fed the same basal diet, supplemented prepartum at 300 g/d per cow with encapsulated fat providing α-linolenic acid (ALA) at 56.1 g/d, and PP at 700 g/d per cow providing 131.0 g/d ALA from FLX. The fat content of the CTL supplement was 99% compared to 80% in the FLX; therefore, the supplemented amounts were different among groups to maintain similar contents of fat in all diets. The fat supplements were specially prepared and supplied by SILA (Venice, Italy). The ingredients and chemical composition of the rations PP, and the profile of the main fatty acids of the supplements were presented previously [17]. The cows were milked thrice daily (at 05:00, 13:00, and 20:00 h), and milk production and BW were recorded automatically at the milking parlor (SAE, Kibbutz Afikim, Israel). Milk samples were collected every two weeks and analyzed for milk fat, protein, lactose and urea by infrared analysis (standard IDF 141C:2000) at the laboratories of the Israeli Cattle Breeders’ Association (Caesarea, Israel). Energy balance was calculated according to NRC (2001) [18]. Blood samples were collected twice a week at 0700 h, and centrifugation was done at 4000 × *g* for 15 min for plasma followed by placing at –80 $^\circ$ C pending analysis. An additional blood sample was collected once a week in EDTA-tubes for RNA extraction.

### Fatty acid composition in plasma

Fatty acid (FA) composition in the plasma at week 1–2 PP was determined in 16 randomly selected cows (8 cows from each group) as described previously [19]. Briefly, the samples were saponified in a mixture of 60% KOH and ethanol, extracted with petroleum ether, and methylated with 5% (v/v) sulfuric acid in methanol. FA methyl esters were analysed with a model 7890 N gas chromatograph (Agilent Technologies, Santa Clara, CA, USA) equipped with a DB-23 capillary column (60 m × 0.25 mm × 0.25 µm; Agilent Technologies) and a flame ionization detector. The initial temperature of the column was set at 130 °C, increased at 6.5 °C/min to 170 °C, and then at 2.75 °C/min to 215 °C, and held at 215 °C for 18 min. Then, the temperature was increased to 230 °C at a rate of 40 °C/min for the remainder of the analysis. The carrier gas was hydrogen, flowing at a linear velocity of 1.6 mL/min; injection volume was 1 µL. Fatty acids were identified according to retention time, as compared to gas chromatograph standard PUFA-2 standard mix (Sigma-Aldrich, St. Louis, MO, USA).

### Analysis of circulating metabolic parameters

Plasma samples (*n* = 13 for CTL and *n* = 11 for the FLX treatment) were analysed for concentrations of non-esterified fatty acids [NEFAs; NEFA C Test Kit (Wako Chemicals GmbH, Neuss, Germany)], β-hydroxybutyric acid [BHBA; Ranbut *D*-3-Hydroxybutyrate kit (Randox, Crumlin, UK)], cortisol (EIA1887, DRG International, Inc., Springfield, NJ, USA), glucose, triglyceride (TG), and aspartate aminotransferase (AST; Cobas C111 Chemistry Analyser, Roche Holding GmbH, Grenzach-Wyhlen, Germany).

### Adrenocorticotrophic hormone administration (ACTH) in-vivo challenge

In order to examine the possible effect of n-3 supplementation on the acute stress response of postpartum cows, we performed an adrenocorticotrophic hormone (ACTH) challenge on d 21 PP according to Trevisi et al. [20]. Briefly, 5 cows were randomly selected from each treatment and were injected with 20 µg of Synacthen (analog for ACTH, Link Pharmaceuticals Ltd, Auckland, New Zealand). Blood samples were taken at six time points; at 5 min before the injection, at the time of the injection, followed by sampling at 15, 30, 45 and 60 min post injection. Cortisol concentrations were measured using an ELISA kit (DRG International Inc., Springfield, NJ, USA).

### PBMC isolation

Peripheral blood mononuclear cells (PBMC), collected during the 1^st^ week PP, were used for proteomic analysis that was previously reported [17]; thus, for immunoblots in the present study we examined PBMC from 5 randomly selected cows from each treatment during the following sampling (on average 17 ± 3 d PP). PBMC were extracted from blood as previously described [17]. Briefly, fresh whole blood sample was diluted at 1:1 ratio using PBS along with phosphatase and protease inhibitors [(10 mmol/L sodium fluoride; 1 mmol/L sodium orthovonadate and 10 mmol/L sodium β-glycerophosphate (Sigma-Aldrich)]. The PBMC were isolated by centrifugation using a Ficoll layer (Histopaque 1077, Sigma-Aldrich). The buffy coat, containing the PBMC, was collected followed by a wash with cold PBS. The viable cells were counted using Trypan blue before bringing them to 1 × 10^7^ cells/mL concentration with PBS containing protease and phosphatase inhibitors (phosphatase inhibitor cocktail, protease inhibitor cocktail, 1% v/v each, Sigma-Aldrich). The cells were frozen at –80 °C for further protein extraction and immunoblotting.

### Subcutaneous adipose tissue biopsy

Subcutaneous AT biopsies from the fat pad around the pin bones were taken from a subset of 6 cows from each treatment that were randomly selected at 7.7 ± 1.9 d PP, as previously described [19]. In short, the biopsy site, a 5 cm × 5 cm area of skin on one side of the pin bone, was prepared by clipping, washing and sterilizing. Cows were sedated with an intramuscular administration of 1 mL of 2% Sedaxylan (xylazine base, 20 mg/mL; Eurovet Animal Health, AE Bladel, the Netherlands). The biopsy site was anesthetized by an 8 mL subcutaneous injection of 2% lidocaine HCl (Esracain 2%, 200 mg per 10 mL; Rafa Laboratories Ltd, Israel). A 1.5 to 2.5 cm scalpel incision was made, under aseptic conditions, through the skin and subcutaneous tissues. In each cow, four samples of approximately 40 mg of fat tissue were captured using tweezers and scissors, tissue samples were then washed with saline followed by snap freeze in liquid nitrogen and then stored at − 80 °C. Additional AT samples that were available were fixed with 4% paraformaldehyde (Sigma-Aldrich) for histology (*n* = 4 from each treatment).

### Liver biopsy

A subset of 5 cows from each treatment group were randomly selected at 10.2 ± 1.0 d PP for liver biopsies procedure as previously described [21]. Briefly, the biopsy was taken from the right side of the animal through the intercostal space. An incision of approximately 1 cm was made through the skin and a few samples were collected under an ultrasound gaudiness using a 14 G × 20 cm needle (Aquila; Pie Medical Imaging BV, Maastricht, the Netherlands Bard Magnum; Bard Biopsy Systems, Tempe, AZ, USA). The liver samples (~ 25 mg each) were snap frozen in liquid nitrogen and stored at − 80 °C pending analysis. The incision site was stapled, treated topically with iodine spray and staples were removed after 7 to 10 d.

### Endocannabinoid measurements

The eCB levels were determined in plasma and liver at the day of the liver biopsy, and in AT samples. Levels of 2-AG, AEA, palmitoylethanolamide (PEA), oleoylethanolamide (OEA) and AA were isolated, purified, and measured by the stable isotope dilution LC–MS/MS method [22].

### Histology and adipocyte analysis

AT samples *(n* = 4 from each treatment) were stained with H&E and imaged with an Olympus BX60 microscope at a magnification of X10 and the Olympus DP73 microscope’s camera (Tokyo, Japan). Two replicates were obtained from each AT sample, and images were analyzed for adipocyte area at 10 × magnification by the Adiposoft plugin (v. 1.15) for ImageJ Fiji (v 2.0.0) as described [23]. Images were spot checked for appropriate quantification. All image capture and analysis were carried out with random identifiers to blind the operator to the treatment of the cow.

### Measurements of hepatic triglycerides and carbohydrates

Liver tissue TG and carbohydrates (CH) were extracted as described by Folch et al. [24] and were quantified using Cobas C111 Chemistry Analyzers (Roche, Basel, Switzerland). Data were normalized according to tissue weight.

### Quantitative real-time PCR

RNA extraction of white blood cells (WBC) was performed by a leukocyte RNA purification kit (NORGEN BioTek Corp, Ontario, Canada) in blood samples collected at 6 ± 3 d PP (*n* = 6 per treatment) using a 2-mL sample of fresh whole blood. Liver (25 mg, *n* = 5 per treatment) and AT (40 mg, *n* = 6 per treatment) samples were homogenized and RNA was extracted using a Tissue Purification Kit (NORGEN BioTek Corp, Ontario, Canada). The RNA purity was assessed using a Nanodrop with 260/280 ratio of above 1.85. First-strand cDNA was generated using a cDNA reverse transcription kit (K1622, RevertAid cDNA Synthesis Kit, Thermo Fisher Scientific, Vilnius, Lithuania). Quantitative real-time PCR was performed using a StepOnePlus instrument (QuantStudio 6 Flex Real-Time PCR System, Applied Biosystems, Foster City, CA, USA) using SYBR green PCR mix (Invitrogen, Carlsbad, CA, USA). In WBC, liver and AT we examined the transcription levels of ECS related genes and inflammatory related genes. In AT and liver samples we also examined gene transcription levels of lipid metabolism and beta-oxidation related genes, the primers of which were chosen according to Chen et al. [25]. All other primers were designed for bovine as described previously [9]. Primers were validated before use and are detailed in Table S1. The WBC were normalized by using two reference genes; Glyceraldehyde-3-Phosphate Dehydrogenase (*GAPDH*) and Beta-Actin (*ACTB*). The liver samples were normalized by using *GAPDH* and Tyrosine 3-Monooxygenase (*YWHAZ*) and the AT was normalized by *GAPDH* and Hypoxanthine Phosphoribosyltransferase 1 (*HPRT1*). The relative quantity of each gene was normalized to the average transcription levels of the reference genes (2^-delta Ct) according to MIQE guidelines [26].

### Immunoblot analysis

Proteins from PBMC, AT and liver were extracted using 5% SDS in 100 mmol/L Tris–HCL lysis buffer containing 1% PMSF, 1% phosphatase inhibitor cocktail and 1% protease inhibitor (Sigma-Aldrich). The samples protein concentrations were measured using the BCA Standard Kit (Cyanagen, Bologna, Italy). Samples were prepared in Laemmli loading buffer (Bio-Rad, Hercules, CA, USA) and resolved by SDS-PAGE under reducing conditions following a transfer to nitrocellulose membrane. The list of antibodies used, their dilutions and source are detailed in Table S2. All samples were normalized by the protein abundance of beta-actin (1:1000, ab46805, Rabbit α Bovine, Abcam Biotech, Cambridge, UK). Goat anti-rabbit HRP-conjugated secondary antibody (Jackson Immunoresearch; 111–035-003, PA, USA) at a concentration of 1:10,000 was used for all proteins except Diacylglycerol lipase alpha (DAGLA), in which the 2^nd^ Ab was Donkey anti Goat HRP-conjugated (1:10,000, ab97110, Abcam biotech). The development was achieved by an ECL reaction for protein detection (Thermo Fisher Scientific, Waltham, MA, USA). Data was processed and analyzed by densitometry using ImageJ software (NIH, Bethesda, MD). To ensure that quantitative data were obtained, chemiluminescence signals were measured during at least five consecutive exposure times to determine each antibody’s linear range of signal intensity. In liver samples, one CTL cow was excluded from analysis due to a technical problem with the protein lysate.

### Statistical analysis

Continues variables such as milk production, feed intake and energy balance, as well as plasma concentrations of metabolites and hormones were analyzed by repeated measurements PROC MIXED, using the following model:

$${Y}_{ijkl}=\mu +{T}_{i}+{C(T)}_{ij}+{DIM}_{ijk}+{E}_{ijkl},$$

where *µ* = overall mean; *T*_*i*_ = treatment effect (_*i*_ = CTL or FLX); *C(T)*_*ij*_ = cow *j* nested in treatment *i*; *DIM*_*ijk*_ = day in lactation as a continuous variable; *E*_*ijkl*_ = random residual.

Data were tested for normality of distribution by the UNIVARIATE procedure of SAS and tested for homogeneity of variance by the Kolmogorov–Smirnov procedure. Because they were not normally distributed, data of milk production, FCM 4%, DMI, NEFA, glucose, BHBA, AST, TG and cortisol were log2 transformed before statistical analysis. The statistical analysis for these parameters was performed on the transformed data, and the least squares mean before transformation are presented in Table 1. The interaction treatment × DIM was tested for all parameters, were not significant, and therefore were removed from the model.

**Table 1:** Milk production, feed intake, energy balance and plasma and liver indices of metabolic state during the first 14 d in lactation of dairy cows supplemented with n-3 fatty acids from flaxseed

Items	**Treatment**^a^		SEM	*P*-value
	**CTL**	**FLX**		
Milk production, kg/d	36.1	33.5	1.35	0.57
Fat corrected milk 4%, kg/d	33.8	33.9	1.54	0.89
Dry matter intake, kg/d	22.2	20.4	0.42	< 0.01
Energy balance, Mcal/d	-1.7	-3.5	1.01	0.22
NEFA^b^, µEq/L	584.4	509.7	69.19	0.80
Glucose, mg/dL	58.9	59.1	1.21	0.95
BHBA^c^, mg/dL	0.6	0.7	0.06	0.16
AST^d^, u/L	959.4	979.2	24.60	0.50
TG^e^, mg/dL	10.8	11.8	0.57	0.32
Cortisol, ng/mL	9.6	5.9	1.17	0.04
Liver TG^e^, mg/g	111.5	100.5	5.60	0.24
Liver CH^f^, mg/g	7.7	5.2	1.37	0.23

^a^Dairy cows were divided into two nutritional regiment groups from −21 to 60 days PP; 1) Control group (CTL)—a standard Israeli diet, 2) FLX—a standard diet supplemented with flaxseed oil containing n-3. ^b^Non-esterified fatty acids; ^c^Beta-hydroxybutyrate; ^d^Aspartate aminotransferase; ^e^Triglycerides; ^f^Carbohydrates. The interaction treatment × DIM were not significant for all data analyzed in this table (except liver TG and CH)

Protein and gene abundances, plasma fatty acid profile, body weight loss, plasma cortisol in response to ACTH challenge, TG and CH in liver were analyzed by SAS GLM (version 9.2, 2002). The distribution of adipocyte sizes and values of eCBs levels in plasma, liver and AT are shown as the mean ± SEM, and were analyzed by unpaired two-tailed Student’s *t*-test.

## Results

### Performance and the metabolic state of the cows

The FLX supplementation altered plasma FA composition; FLX increased the plasma percentage of alpha-linolenic acid (C18:3n-3) compared to control cows (3.9% and 1.8 in FLX and CTL, respectively, SEM = 0.15, *P* < 0.0001), and the total n-3 FA percentage in plasma was higher in the FLX cows (4.9% vs. 2.6 in FLX and CTL, respectively, SEM = 0.17, *P* < 0.0001). The n-6/n-3 FA ratio in plasma tended to decrease in the FLX compared to CTL (24.2 and 9.8 in CTL and FLX respectively, SEM = 5.32, *P* = 0.07). The full FA profile in plasma is presented in Table S3. Milk production, feed intake, energy balance and plasma indices of metabolic state during the first 14 d in lactation are presented in Table 1. Milk yields (*P* = 0.57) and fat corrected milk 4% (*P* = 0.89) were similar among groups, while the average dry matter intake was 8.1% lower in the FLX than in the CTL (*P* = 0.006), and the average calculated energy balance was not different between groups (*P* = 0.22; Table 1). The body weight (BW) loss between week 1 and week 3 PP was not different between groups (36.5 vs. 31.0 kg in CTL and FLX, respectively, SEM = 3.97, *P* = 0.34). During the first 14 d PP, average concentrations of NEFA, glucose, BHBA, AST, and TG did not differ between groups, while plasma cortisol was lower in FLX than in CTL (*P* = 0.04; Table 1). However, in response to an ACTH challenge at 21 d PP, average plasma cortisol concentrations were not different between CTL and FLX cows (39.4 and 40.3 ng/mL, in CTL and FLX, respectively, SEM = 5.24, *P* = 0.90; Fig. S1).

### Effects of dietary n-3 on ECS components in blood

As shown in Table 2, at 10 d PP lower plasma levels of AEA (by 49.7%; *P* = 0.03) and AA (by 44.2%; *P* = 0.02) were found in FLX compared to CTL, while levels of 2-AG, PEA and OEA were not different between groups. In WBCs during the 1^st^ week PP, the relative gene transcription levels of cannabinoid receptor 1 (*CNR1*), but not cannabinoid receptor 2 (*CNR2*) or *MGLL*, tended to be lower (by 2.5 folds; *P* = 0.10) in the FLX compared to CTL (Table S4). In PBMCs, the average protein abundance of CB1 and CB2 were not different between treatments, but the abundance of MGLL was higher in FLX than in CTL (0.835 vs. 1.394 AU in CTL and FLX, respectively, SEM = 0.16, *P* = 0.04, Fig. S3).

**Table 2:** eCB concentrations in plasma, liver and AT samples of PP dairy cows supplemented with n-3 fatty acids from flaxseed

Items	**Treatment**^a^		**SEM**	***P*-value**
	**CTL**	**FLX**		
Plasma				
2-AG^b^, fmol/mL	10.5	9.1	2.13	0.65
AA^c^, pmol/mL	581.2	324.3	61.18	0.02
AEA^d^, fmol/mL	232.2	116.8	31.82	0.03
PEA^e^, fmol/mL	0.4	0.0	0.31	0.33
OEA^f^, fmol/mL	34.1	34.3	9.66	0.99
Adipose				
2-AG^b^, fmol/mg	97.0	69.6	25.90	0.48
AA^c^, pmol/mg	1.3	1.0	0.16	0.24
AEA^d^, fmol/mg	0.7	0.7	0.17	0.97
PEA^e^, fmol/mg	16.1	6.2	2.53	0.02
OEA^f^, fmol/mg	125.2	100.2	15.90	0.30
Liver				
2-AG^b^, fmol/mg	287.6	497.8	72.03	0.07
AA^c^, pmol/mg	6.9	8.2	1.65	0.59
AEA^d^, fmol/mg	0.7	0.9	0.40	0.73
PEA^e^, fmol/mg	13.9	10.6	8.17	0.78
OEA^f^, fmol/mg	44.2	15.9	15.16	0.22

^a^Dairy cows were divided into two nutritional regiment groups from −21 to 60 days PP; 1) Control group (CTL)—a standard Israeli diet, 2) FLX—a standard diet supplemented with flaxseed oil containing n-3. ^b^2-Arachidonoylglycerol; ^c^Arachidonic acid; ^d^Anandamide; ^e^*N*-palmitoylethanolamine; ^f^ *N*-Oleoylethanolamine. Liver samples were taken from 5 cows per treatment at 10.2 ± 1.0 DIM while AT samples were obtained from 6 cows from each treatment at 7.7 ± 1.9 DIM

### Effects of dietary n-3 on ECS components in adipose tissue

**Fig. 1:**
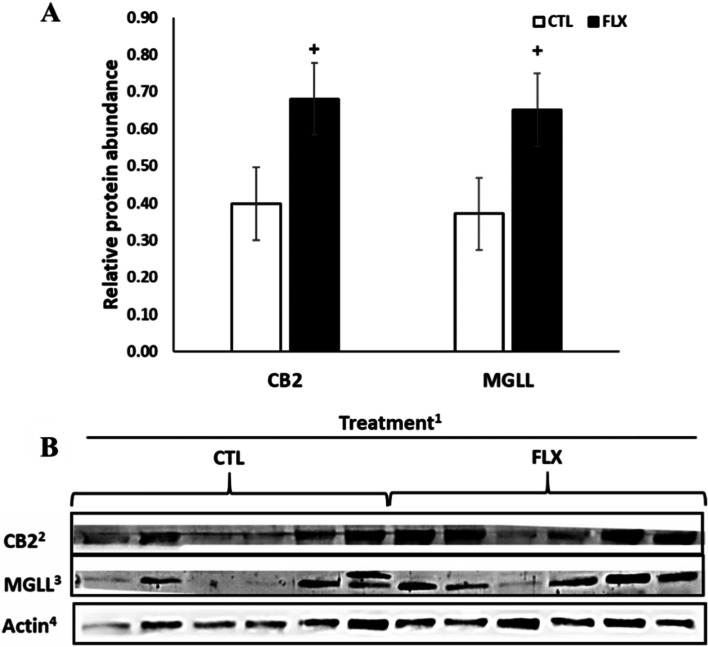
Adipose tissue relative protein abundance of ECS-related proteins of PP dairy cows supplemented with n-3 fatty acids. ^+^*P* ≤ 0.10. Dairy cows were divided into two nutritional regiment groups from −21 to 60 days PP; 1) Control group (CTL)—a standard Israeli diet, 2) FLX—a standard diet supplemented with flaxseed oil containing n-3. ^2^Cannabinoid receptor 2; ^3^Monoglyceride lipase; ^4^β-Actin which was used as reference protein. *n* = 6 per treatment

In the AT collected at 7 d PP, the PEA levels decreased by 61.5% in the FLX compared to the CTL (*P* = 0.02), while no differences were observed in the levels of 2-AG, AA, AEA, and OEA between groups (Table 2). The transcription levels of genes related to lipid metabolism and FA β-oxidation were higher in FLX vs. CTL: the average relative transcription level of Lipase E, hormone sensitive type (*LIPE*; by 7.5 folds; *P* = 0.02), Carbohydrate response element binding protein (*MLXIPL;* by 3.8 folds; *P* = 0.01), Acyl-CoA oxidase (*ACOX1*; by 4.1 folds; *P* < 0.001), Sterol regulatory element binding transcription factor 1 (*SREBP-1;* by 2.1 folds; *P* = 0.01) and Peroxisome proliferator activated receptor gamma (*PPARG*; by 2.6 folds; *P* = 0.04) were higher in FLX than in CTL, and Acyl-CoA synthetase long chain family member 1 (*ACSL1*) tended to be higher in FLX than in CTL (by 2.4 folds; *P* = 0.07; Table 3). As shown in Fig. 1, the protein abundance of CB2 in AT tended to be higher in FLX compared to CTL (*P* = 0.08), and MGLL tended to be higher in FLX compared to CTL (*P* = 0.10). The protein abundances of CB1, DAGLA and FAAH in AT did not differ among groups (Table S5). In addition, the average protein abundance of nuclear factor kappa-light-chain-enhancer of activated B cells (NFĸB), tumor necrotizing factor alpha (TNF-α), interlukin-10 (IL-10), hormone sensitive lipase (HSL), and fatty acids synthase (FASN) were not different between groups (Table S5). Analyzing the distribution of adipocytes from different sizes demonstrated a tendency for a higher percentage of relatively large (4001–6000 µm^2^) adipocytes in FLX compared to CTL (*P* = 0.07), while other cell sizes were similarly distributed among groups (Fig. S2).

**Table 3:** Adipose tissue average gene transcription levels (relative quantities; RQ) of ECS-related, lipid metabolism and inflammatory genes of PP dairy cows supplemented with n-3 fatty acids

Genes	**Treatment**^a^		SEM	*P*-value
	**Control**	**FLX**		
Endocannabinoid-related				
*CNR1*^b^	1.65	1.71	0.090	0.672
*CNR2*^c^	0.34	0.30	0.023	0.249
*MGLL*^d^	0.68	0.76	0.082	0.547
*NAPEPLD*^e^	0.02	0.03	0.004	0.120
*FAAH*^f^	0.07	0.08	0.033	0.897
Lipid metabolism and beta oxidation				
*PPARA*^g^	0.08	0.13	0.022	0.217
*PPARG*^h^	0.52	1.35	0.246	0.039
*LIPE*^i^	1.67	12.46	2.894	0.025
*FASN*^j^	0.80	1.97	0.577	0.185
*FABP4*^k^	61.18	147.02	42.787	0.186
*SREBP1*^l^	0.35	0.73	0.093	0.015
*MLXIPL*^m^	0.04	0.15	0.027	0.015
*ACOX1*^n^	0.13	0.53	0.060	< 0.001
*ACSL1*^o^	0.26	0.62	0.128	0.077
*CPT1A*^p^	0.02	0.09	0.034	0.144
*CPT2*^q^	0.76	1.00	0.122	0.205
Inflammation				
*TNFA*^r^	0.005	0.003	0.001	0.227
*IL1B*^s^	0.02	0.01	0.010	0.296
*IL6*^t^	0.60	0.57	0.077	0.835
*NFKB1*^u^	0.06	0.11	0.034	0.327
*TLR4*^v^	0.15	0.12	0.03	0.496
*CD14*^w^	0.55	0.52	0.05	0.706
*PTGS2*^x^	0.68	0.76	0.082	0.547
*PLA2*^y^	0.36	0.29	0.067	0.489
*IL10*^z^	0.007	0.009	0.002	0.480

^a^Dairy cows were divided into two nutritional regiment groups from −21 to 60 days PP; 1) Control group (CTL)—a standard Israeli diet, 2) FLX—a standard diet supplemented with flaxseed oil containing n-3. ^b^Cannabinoid-1 receptor; ^c^Cannabinoid-2 receptor; ^d^Monoglyceride lipase; ^e^N-Acyl phosphatidylethanolamine phospholipase D; ^f^Fatty acid amide hydrolase; ^g^Peroxisome proliferator activated receptor alpha; ^h^Peroxisome proliferator activated receptor gamma; ^i^Lipase E, hormone sensitive type; ^j^Fatty acid synthase; ^k^Fatty acid binding protein 4; ^l^Sterol regulatory element binding transcription factor 1; ^m^Carbohydrate response element binding protein; ^n^Acyl-CoA oxidase; ^o^Acyl-CoA synthetase long chain family member 1; ^p^Carnitine palmitoyltransferase 1; ^q^Carnitine palmitoyltransferase 2; ^r^Tumor necrosis factor α; ^s^Interleukin 1 beta; ^t^Interleukin 6; ^u^Nuclear factor kappa-light-chain-enhancer of activated B cells; ^v^Toll like receptor 1; ^w^CD14 molecule 10; ^x^Prostaglandin endoperoxide synthase 2; ^y^Phospholipase A2 group IIA; ^z^Interleukin 10

### Effects of dietary n-3 on ECS components in liver

**Fig. 2:**
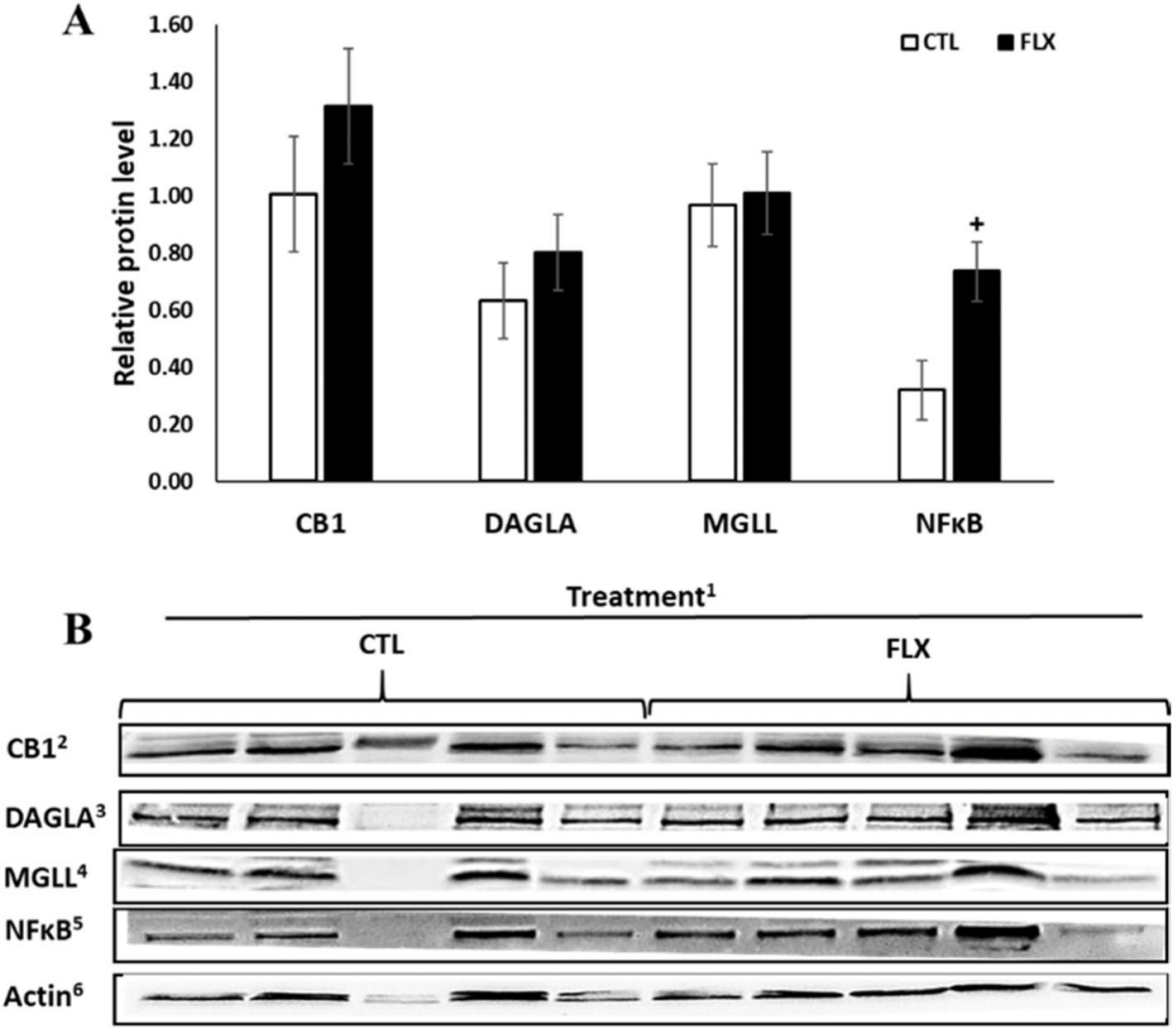
Liver tissue relative protein abundance of ECS- related protein and inflammatory protein of PP dairy cows supplemented with n-3 fatty acids. ^+^*P* = 0.1.^1^Dairy cows were divided into two nutritional regiment groups from −21 to 60 days PP; 1) Control group (CTL)—a standard Israeli diet, 2) FLX—a standard diet supplemented with flaxseed oil containing n-3. ^2^Cannabinoid receptor 1; ^3^Diacylglycerol lipase alpha; ^4^Monoglyceride lipase; ^5^Nuclear factor kappa-light-chain-enhancer of activated B cells; ^6^β-Actin which was used as reference protein. *n* = 5 per treatment. As mentioned in text, sample number 3 from the control group was an outlier in protein amount, therefore it was excluded from analysis

In the liver, the average levels of 2-AG tended to increase in FLX compared to the control (by 73.1%; *P* = 0.07), but the levels of AA, AEA, PEA and OEA did not differ among groups (Table 2). No differences were found in the levels of triglycerides or carbohydrate concentration in the liver (Table 1). As shown in Table 4, the relative gene transcription levels of *CNR1* (1.7 folds; *P* = 0.11) and *CNR2* (1.5 folds; *P* = 0.07) in liver tended to be lower in the FLX compared to the CTL, whereas no changes were observed in the gene transcription of *MGLL,* N-Acyl phosphatidylethanolamine phospholipase D *(NAPEPLD*) and *FAAH*. A 1.4-fold decrease in pro-inflammatory *IL6* was found in the FLX compared to the control (1.5 folds; *P* = 0.03), while no significant changes were neither documented in the inflammatory genes *TNFA, IL1B, NFKB1, SAA2, Hp* and *IL10* nor in the lipid metabolism genes *PPARA, SREBP1, MLXIPL, ACOX1, ACSL1, FABP1, CPT1A,* and *CPT2* among treatments (Table 4). As shown in Fig. 2, the average protein abundance of NFĸB in the liver tended to increase in the FLX compared to CTL (*P* = 0.06), while the abundances of CB1, DAGLA and MGLL were not different between groups. Average liver protein abundances of FAAH, IL-10 and TNF-α were also not different between treatments (Table S6).

**Table 4:** Liver average gene transcription levels (relative quantities; RQ) of ECS-related, lipid metabolism and inflammatory genes of PP dairy cows supplemented with n-3 fatty acids

Genes	**Treatment**^a^		SEM	*P*-value
	**Control**	**FLX**		
Endocannabinoid-related				
*CNR1*^b^	0.05	0.03	0.007	0.11
*CNR2*^c^	0.71	0.46	0.083	0.07
*MGLL*^d^	2.65	2.24	0.528	0.60
*NAPEPLD*^e^	0.06	0.07	0.006	0.49
*FAAH*^f^	1.71	1.78	0.240	0.85
Lipid metabolism and beta oxidation				
*PPARA*^g^	6.76	9.17	1.620	0.32
*SREBP1*^h^	1.22	1.26	0.135	0.87
*MLXIPL*^i^	1.45	1.64	0.310	0.67
*ACOX1*^j^	29.39	35.70	5.074	0.41
*ACSL1*^k^	18.28	13.74	3.413	0.37
*FABP1*^l^	83.21	116.75	16.969	0.20
*CPT1A*^m^	1.12	0.42	0.371	0.22
*CPT2*^n^	8.89	7.87	1.432	0.63
Inflammation				
*TNFA*^o^	0.02	0.03	0.005	0.24
*IL1B*^p^	0.06	0.07	0.014	0.81
*IL6*^q^	0.27	0.18	0.024	0.03
*NFKB1*^r^	0.18	0.22	0.031	0.46
*SAA2*^s^	65.99	28.32	26.738	0.35
*HP*^t^	38.42	10.17	12.899	0.16
*IL10*^u^	0.01	0.01	0.003	0.53

^a^Dairy cows were divided into two nutritional regiment groups from −21 to 60 days PP; 1) Control group (CTL)—a standard Israeli diet, 2) FLX—a standard diet supplemented with flaxseed oil containing n-3. ^b^Cannabinoid-1 Receptor; ^c^Cannabinoid-2 receptor; ^d^Monoglyceride lipase; ^e^N-Acyl phosphatidylethanolamine phospholipase D; ^f^Fatty acid amide hydrolase; ^g^Peroxisome proliferator activated receptor alpha; ^h^Sterol regulatory element binding transcription factor 1; ^i^Carbohydrate response element binding protein; ^j^Acyl-CoA oxidase; ^k^Acyl-CoA synthetase long chain family member 1; ^l^Liver fatty acid-binding protein; ^m^Carnitine palmitoyltransferase 1; ^n^Carnitine palmitoyltransferase 2; ^o^Tumor necrosis factor α; ^p^Interleukin 1 beta; ^q^Interleukin 6; ^r^Nuclear factor kappa-light-chain-enhancer of activated B cells; ^s^Serum amyloid A2; ^t^Haptoglobin; ^u^Interleukin 10

## Discussion

In the present study, we hypothesized that supplementing peripartum cows with n-3 fatty acids will lower the systemic availability of AA and reduce ECS activation in blood, liver and AT. Indeed, we found lower AA and AEA levels in plasma of FLX cows, along with lower PEA in AT and a tendency for higher 2-AG levels in the liver of FLX compared to CTL. This was accompanied by moderate changes in gene and protein abundances of ECS components in FLX tissues, such as a tendency for decreased *CNR1* and *CNR2* gene transcription levels in the liver, and a tendency for increase in CB2 protein expression in the AT. Supplementation of n-3 was hypothesized to lower ECS activation, thus possibly reduce feed intake, as in mammals CB1 activation in the brain increases feed intake [27]. Although we did not examine ECS components in the brain in this study, we have found a reduction in feed intake, which could be related to a certain alteration in the activation of the ECS in the FLX cows during the early PP period. This could be supported by the tendency for decreased gene transcription of the CB1 receptor in both WBC and liver samples, which could possibly suggest a systemic reduction in ECS activity that was related to the lower intake. However, other studies in which n-3 from flaxseed was supplemented to peripartum cows showed increased intake compared with control cows [28], while others observed no effect of flaxseed supplementation on intake [29–31], or reduced intake [32]; possible causes for these inconsistencies were described in [33]. Nevertheless, our findings support the hypothesis that lower ECS activation may be related to lower feed intake in PP dairy cows.

We found a tendency toward lower gene transcription levels of CB1 in WBC along with a higher protein abundance of MGLL in PBMC of FLX compared to CTL blood, although it was not translated into lower plasma 2-AG levels. In addition to the main role of the ECS as a regulator of energy metabolism, it is also involved in maintaining a balanced inflammatory and redox state [34]. The effects of ECS activation on inflammatory responses are complex and vary depending on the tissue, eCBs, and the type of receptor. For example, AEA has anti-inflammatory effects, including the inhibition of chemoattractant cytokines secretion, especially those released at the early stages of the inflammatory process such as IL-6, IL-8, and MCP-1, along with completely blocking lipopolysaccharide (LPS)-triggered activation of NFkB pathway in periodontal tissues [35]. As for the receptors, activation of the CB1 suppresses the proliferation of cells of the adaptive immune system, especially T-cells [36]. There are reports indicating that inhibition of ECS receptor activity causes inflammatory responses [37]. We have previously reported an enrichment of the acute-phase-signaling and complement pathways in PBMCs from FLX compared to CTL [17], and based on the moderate changes in CB1 and MGLL in WBC, we suggest that nutritional supplementation of n-3 might affect the inflammatory response of these cells via the ECS, however this premise requires further examination.

A reduction in ECS activation should coincide with increased lipolysis; however, we did not observe differences in body weight loss, plasma NEFAs and EB, and we found a tendency for a higher proportion of large adipocytes in AT of FLX compared to CTL, which does not support the hypothesis of increased lipolysis in these cows. We observed increased transcription levels of several lipid metabolism genes (*FABP4*, *MLXIPL*, *ACOX1*) in FLX AT, possibly suggesting increased lipogenesis in AT of cows supplemented with n-3; at the same time, we also found higher transcription levels of the main lipolytic enzyme LIPE. In rodent and human adipocytes, CB1 receptor stimulation increases the uptake of glucose and lipogenesis while inhibiting lipolysis [38]. CB1 conducts its response via G protein Gi/o-mediated reduction in adenylate cyclase action, suppressing the activation of HSL through the halt in cyclic adenosine monophosphate (cAMP) production [39]. Higher gene transcription levels of *CNR2, NAPEPLD*, and *FAAH* in AT coincides with enhanced transcription of pro-inflammatory genes (*TNFA, IL6, IL1B*) at 21 and 42 d PP in cows exhibiting intense AT lipolysis [12]. The anti-lipolytic effect of CB1 stimulation in adipocytes occurs through Gi/o inhibition of cAMP production, which limits the downstream phosphorylation of HSL and perilipin [40]. Transcriptional effects downstream of CB1 activation in AT include the suppression of lipolysis-associated enzymes (carnitine-acyl-CoA transferase, carnitine palmitoyltransferase 2, and crotonase), along with downregulation of β-adrenergic and growth hormone receptor expression [41]. CB1 stimulation in AT sympathetic nerves suppresses catecholamine release, subsequently downregulating HSL activity through the decrease in cAMP produced by the AT [42]. Our findings may indicate of increased lipid metabolism, possibly promoting lipogenesis and thus limiting lipolysis in AT of FLX cows despite lower feed intake.

We found a tendency towards increased abundance of CB2 in AT of FLX cows. CB2 is found primarily in microvascular endothelial cells and on the surface of immune cells, most commonly those derived from the hematopoietic lineage [43]. CB2 is known to exert anti-inflammatory effects in peripheral tissues [43], and only low levels of CB2 expression are detected in monogastric AT [44]. In humans, CB2 expression has been shown to decrease as pre-adipocytes mature into adipocytes in vitro [45]. This gene transcription pattern suggests that within AT, CB2 content is higher in pre-adipocytes, macrophages, and vascular cells rather than in mature adipocytes [45]. Therefore, we assume that the increased CB2 in AT could suggest of a higher presence of immune cells in the AT of the FLX cows.

**Fig. 3:**
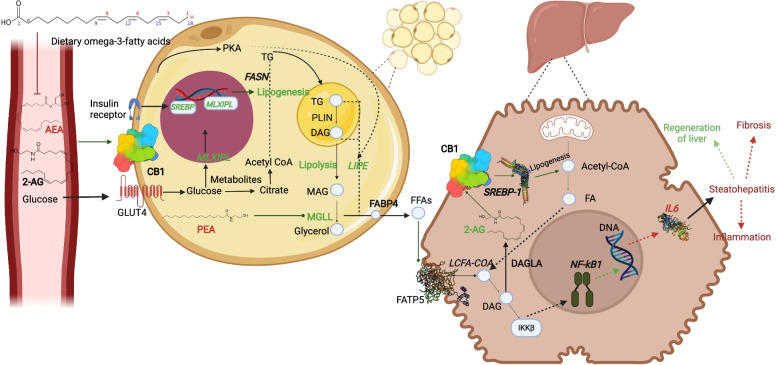
Proposed model of effects of n-3 supplementation on the ECS in blood, liver and adipose tissue in peripartum dairy cows. n-3 supplementation reduces plasma AEA through reduced AA, this may result in reduction of CB1 receptor gene transcription levels in WBCs and liver. In AT, the increased *MLXIPL* and *SREBP* gene transcription may lead to increased lipogenesis. Increased *LIPE* and MGLL could result in the release of more FAs from the AT. These FAs can move to the liver and be converted to diacylglycerols (DAGs) by long-chain fatty acyl-CoA (LCFA-coA). In the liver, DAGs are converted to 2-AG by diacylglycerol lipase alpha (DAGLA). DAGs may activate through IkB kinase (IKKB) signalling pathway that can induce nuclear factor kappa B subunit 1 (*NFKB1*) gene transcription. The decrease in *IL6* transcription levels may possibly lead to reduced inflammation and high regeneration of the liver. The green colour marks factors that were increased in the present study, while the Red marks factors that have been decreased. Genes are represented in italics. AEA: Anandamide; 2-AG: 2-Arachidonoylglycerol; CB1: Cannabinoid receptor 1; GLUT4: Glucose transporter type 4; SREBP: Sterol regulatory element binding transcription factor; MLXIPL: Carbohydrate response element binding protein; PEA: N-palmitoylethanolamine; TG: Triglycerides; FASN: Fatty acid synthase; PLIN: Perilipin; DAG: Diacylglycerol; LIPE: Lipase, hormone-sensitive; MAG: Monoacylglycerol; MGLL: Monoglyceride lipase; FABP4: Fatty acid binding protein 4; FFAs: Free fatty acids; LCFA-COA: long-chain fatty acids (LCFAs) esterification with coenzyme A (CoA); DAGLA: Diacylglycerol lipase alpha; IKKβ: IκB kinase β; NF-κB1: Nuclear factor NF-kappa-B p105 subunit; IL6: Interleukin 6

In liver, we found that the mRNA transcription of CB1 and CB2 tended to be lower in the liver of FLX cows, while 2-AG tended to be higher and the transcription of *IL6* was reduced in liver of FLX cows, which could indicate of reduced inflammation in their liver; although the abundance of NFKB tended to be higher in liver of FLX compared to CTL. The higher 2-AG in liver might indicate of a local synthesis or accumulation of eCBs in hepatocytes, possibly in response to lower gene transcription levels of CB1 and CB2, however this requires further investigation. Together, dietary n-3 had a moderate effect on some ECS components in the liver of PP cows; it is suggested that the higher 2-AG could be a local response to the systemic reduction in ECS tone and may be related to a possible lower inflammatory tone in the liver, however this requires further investigation. Recently, a study in mice compared a large range of eCBs in liver and subcutaneous AT, and related them to the inflammatory state of the tissues; interestingly, they reported of a tissue-dependent relationship between the eCBs and inflammation [46]. In line with this premise, in the present study we found different responses of ECS and inflammatory parameters in AT and liver (summarized in Fig. 3), supporting the tissue-dependent responses in this regard.

## Conclusions

Dietary supplementation of n-3 fatty acids reduced plasma levels of AA and AEA, and tended to reduce gene transcription of *CNR1* in WBC and liver during the early PP period compared to controls, indicating a possible reduction in ECS activation that could be related to the decreased feed intake. However, similar body weight loss, plasma NEFAs, energy balance, and increased cell size in AT of FLX do not support increased lipolysis in these cows. Nonetheless, some moderate alterations in ECS components were evident in blood, liver and AT, possibly indicating of increased lipid metabolism in AT and lower inflammation in liver, as shown in our proposed model in Fig. 3. Together, our findings demonstrate that nutritional supplementation of n-3 FA has a moderate effect on ECS components and may be related to the tissue-dependent metabolic and inflammatory responses in AT and liver of peripartum dairy cows.

## Supplementary Information

**Additional file 1: Fig. S1.** Plasma cortisol concentrations in response to ACTH-challenge of postpartum dairy cows supplemented with n-3 (21 DIM). **Fig. S2.** Distribution of adipocytes according to adipocyte area of adipose tissue of PP dairy cows supplemented with n-3 fatty acids or control diet. **Fig. S3.** Peripheral blood mononuclear cells (PBMC) protein abundance of ECS-related proteins during the 1^st^ week PP of dairy cows supplemented with n-3 fatty acids. **Fig. S4.** Western blot images of Adipose tissue average protein expression of ECS-related, lipid metabolism and inflammatory proteins of PP dairy cows supplemented with n-3 fatty acids. **Fig. S5.** Western blot images of Liver average protein expression of ECS-related, lipid metabolism and inflammatory proteins of PP dairy cows supplemented with n-3 fatty acids. **Table S1.** List of primers used to determine gene transcription levels. **Table S2.** List of immunoblot Ab's used to measure protein abundance. **Table S3.** Fatty acid (FA) profile in plasma of postpartum dairy cows supplemented with n-3. **Table S4.** White blood cells (WBC) average gene transcription levels (relative quantities; RQ) of ECS-related genes during the 1^st^ week PP of dairy cows supplemented with n-3 fatty acids. **Table S5.** Adipose tissue relative protein abundance of ECS-related, lipid metabolism and inflammatory proteins of PP dairy cows supplemented with n-3 fatty acids. **Table S6.** Liver tissue relative protein abundance of ECS-related, lipid metabolism and inflammatory proteins of PP dairy cows supplemented with n-3 fatty acids.

## Data Availability

All data generated or analysed during this study are included in this published article and its supplementary information files.

## Abbreviations

2-AG: 2-Arachidonoylglycerol
AA: Arachidonic acid
ACTH: Adrenocorticotrophic hormone
AEA: Anandamide
ALA: α-Linolenic acid
AST: Aspartate aminotransferase
AT: Adipose tissue
BHBA: β-Hydroxybutyric acid
BW: Body weight
CB1: Cannabinoid receptor 1
CB2: Cannabinoid receptor 2
CH: Carbohydrates
eCBs: Endocannabinoids
ECS: Endocannabinoid system
FA: Fatty acid
FAAH: Fatty acid amide hydrolase
MGLL: Monoacylglycerol lipase
n-3: Omega-3
n-6: Omega-6
NEFAs: Non-esterifies fatty acids
OEA: Oleoylethanolamide
PBMC: Peripheral blood mononuclear cells
PEA: Palmitoylethanolamide
PP: Postpartum
TG: Triglycerides
WBC: White blood cells
