# Supplementary material for: Effects of omega-3 supplementation on components of the endocannabinoid system and metabolic and inflammatory responses in adipose and liver of peripartum dairy cows
Source: J Anim Sci Biotechnol. 2022 Oct 2;13:114. doi: 10.1186/s40104-022-00761-9 (PMC9526899; doi:10.1186/s40104-022-00761-9)
Supplement: Supplementary file 1 — Additional file 1: Fig. S1. Plasma cortisol concentrations in response to ACTH-challenge of postpartum dairy cows supplemented with n-3 (21 DIM). Fig. S2. Distribution of adipocytes according to adipocyte area of adipose tissue of PP dairy cows supplemented with n-3 fatty acids or control diet. Fig. S3. Peripheral blood mononuclear cells (PBMC) protein abundance of ECS-related proteins during the 1st week PP of dairy cows supplemented with n-3 fatty acids. Fig. S4. Western blot images of Adipose tissue average protein expression of ECS-related, lipid metabolism and inflammatory proteins of PP dairy cows supplemented with n-3 fatty acids. Fig. S5. Western blot images of Liver average protein expression of ECS-related, lipid metabolism and inflammatory proteins of PP dairy cows supplemented with n-3 fatty acids. Table S1. List of primers used to determine gene transcription levels. Table S2. List of immunoblot Ab's used to measure protein abundance. Table S3. Fatty acid (FA) profile in plasma of postpartum dairy cows supplemented with n-3. Table S4. White blood cells (WBC) average gene transcription levels (relative quantities; RQ) of ECS-related genes during the 1st week PP of dairy cows supplemented with n-3 fatty acids. Table S5. Adipose tissue relative protein abundance of ECS-related, lipid metabolism and inflammatory proteins of PP dairy cows supplemented with n-3 fatty acids. Table S6. Liver tissue relative protein abundance of ECS-related, lipid metabolism and inflammatory proteins of PP dairy cows supplemented with n-3 fatty acids. [file 40104_2022_761_MOESM1_ESM.docx]

**Supplementary Figures and Tables**

**Fig S1.** Plasma cortisol concentrations in response to ACTH-challenge of postpartum dairy cows supplemented with n-3 (21 DIM). The cows were divided into two nutritional groups from –21 to 60 days PP; 1) Control group (CTL)—a standard lactating diet, 2) Flaxseed (FLX)—a standard diet supplemented with encapsulated flaxseed oil enriched with C18:3n-3. Synacthen- an analog to ACTH

**Fig S2.** Distribution of adipocytes according to adipocyte area. Adipose tissue of PP dairy cows supplemented with n-3 fatty acids or control diet. Dairy cows were divided into two nutritional groups from 21 d before expected calving to 60 days PP; 1) Control group (CTL)—a standard lactating diet, 2) FLX—a standard lactating diet supplemented with flaxseed oil containing n-3. **^+^***P* = 0.07


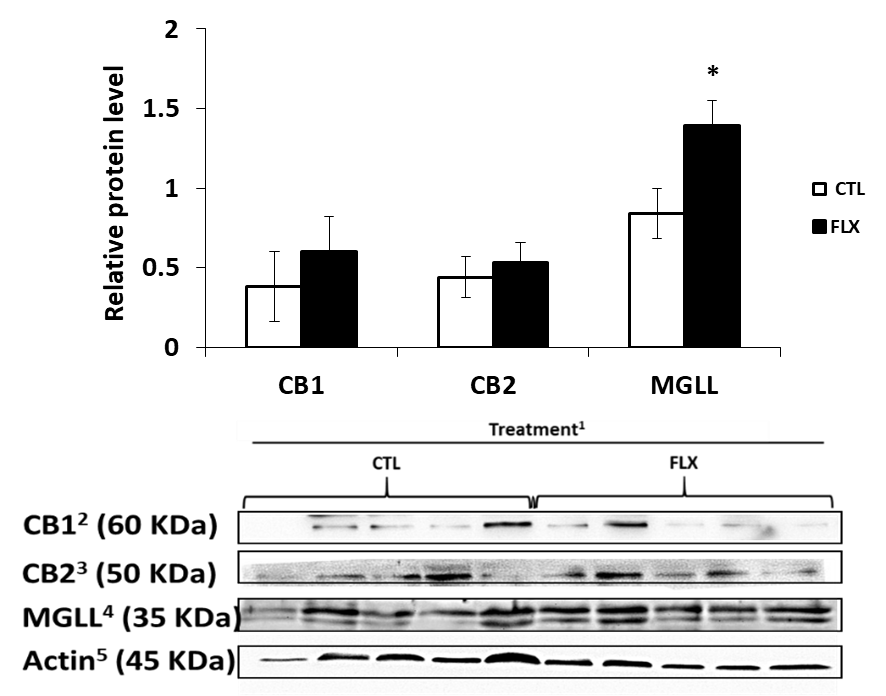


**Fig. S3.** Peripheral blood mononuclear cells (PBMC) protein abundance of ECS-related proteins during the 1st week PP of dairy cows supplemented with n-3 fatty acids. *P* < 0.05. ^1^Dairy cows were divided into two nutritional regiment groups from –21 to 60 days PP; 1) Control group (CTL)—a standard Israeli diet, 2) FLX—a standard diet supplemented with flaxseed oil containing n-3. *n*=5 per treatment. ^2^Cannabinoid receptor 1; ^3^Cannabinoid receptor 2; ^4^ Monoglyceride lipase; ^5^β-Actin which was used as reference protein

***
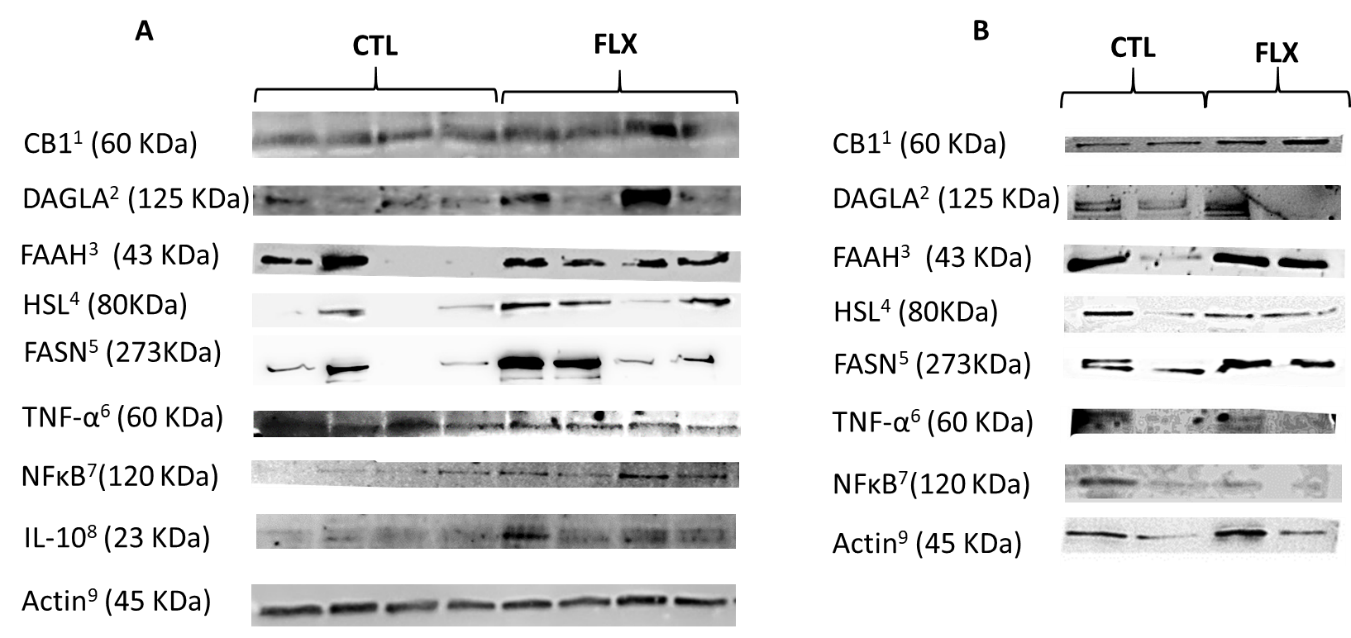
***

**Fig. S4.** Western blot images of adipose tissue average protein expression of ECS-related, lipid metabolism and inflammatory proteins of PP dairy cows supplemented with n-3 fatty acids. Dairy cows were divided into two nutritional regiment groups from –21 to 60 days PP; 1) Control group (CTL)­—a standard Israeli diet, 2) FLX—a standard diet supplemented with flaxseed oil containing n-3. A: 1^st^ set of samples, *n*=4 per treatment; B: 2^nd^ set of samples, *n*=2 per treatment. ^1^Cannabinoid receptor 1; ^2^Diacylglycerol lipase alpha; ^3^Fatty acid amide hydrolase; ^4^Hormone sensitive lipase; ^5^Fatty acid synthase; ^6^Tumor necrosis factor α; ^7^Nuclear factor kappa-light-chain-enhancer of activated B cells; ^8^Interleukin 10; ^9^All samples were corrected by the β-Actin protein expression levels

***
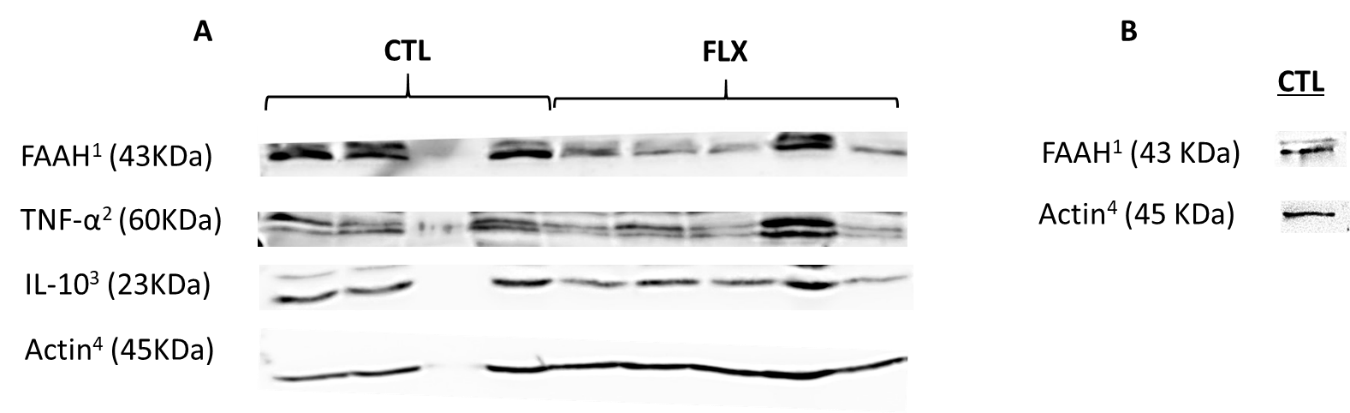
***

**Fig. S5.** Western blot images of liver average protein expression of ECS-related, lipid metabolism and inflammatory proteins of PP dairy cows supplemented with n-3 fatty acids. Dairy cows were divided into two nutritional regiment groups from –21 to 60 days PP; 1) Control group (CTL)—a standard Israeli diet, 2) FLX—a standard diet supplemented with flaxseed oil containing n-3. A: 1^st^ set of samples, *n*=4 for CTL and *n*=5 for FLX treatment; B: 2^nd^ set of samples *n*=1 of CTL treatment. ^1^Fatty acid amide hydrolase; ^2^Tumor necrosis factor α; ^3^Interleukin 10; ^4^All samples were corrected by the β-Actin protein expression levels

**Table S1**. List of primers used to determine gene transcription levels

| Gene | Description | GenBank accession no. | Sequence 5`→3` (Forward, Reverse) |
| --- | --- | --- | --- |
| *GAPDH* | Glyceraldehyde-3-phosphate dehydrogenase | NM_001034034.2 | F:GATTGTCAGCAATGCCTCCT  R:GGTCATAAGTCCCTCCACGA |
| *ACTB* | Beta- actin | AY_141970.1 | F: CTCTTCCAGCCTTCCTTCCT  R: AGAGGTCCTTGCGGATGTC |
| *YWHAZ* | Tyrosine 3-monooxygenase/tryptophan 5-monooxygenase activation protein zeta | NM_174814.2 | F:GGAGGGTCGTCTCCAGTATTGA  R:CGAGCCATCTGCTGTTTTTTC |
| *HPRT1* | hypoxanthine Phosphoribosyltransferase 1 | NM_001034035.2 | F:CACTGGGAAGACATGCAGA  R: ACACTTCGAGGGGTCCTTTT |
| *CNR1* | Cannabinoid receptor 1 | NM_001242341.2 | F:AAGCCCGCATGGACATTAGGTTAG  R: AGCAGAGGGCCCCAGCAGAT |
| *CNR2* | Cannabinoid receptor 2 | NM_001192303.1 | F: TCTTCGCCGGCATCATCTAC  R: CATCCGGGCTATTCCAGACA |
| *MGLL* | Monoglyceride lipase | NM_001206681.1 | F: GCAACCAGCTGCTCAACAC  R: AGCGTCTTGTCCTGGCTCTT |
| *FAAH* | Fatty acid amide hydrolase | NM_001099102.2 | F: TTCCTGCCAAGCAACATACCT  R: CACGAAATCACCTTTGAAGTTCTG |
| *NAPEPLD* | N-Acyl phosphatidylethanolamine phospholipase D | NM_001015680.1 | F: AGAGATCACAGCAGCGTTCCAT  R: ACTCCAGCTTCTTCAGGGTCATC |
| *DAGLA* | Diacylglycerol lipase alpha | NM_001192583.3 | F: GGCTCAAAGTGTTCCTCTGC  R: TGTCCAGGTCTCGGAAAAAC |
| *PPARA* | Peroxisome proliferator activated receptor alpha | NM_001034036.1 | F: CCCTCTTTGTGGCTGCTATC  R: GCACAATACCCTCCTGCATT |
| *PPARG* | Peroxisome proliferator activated receptor gamma | NM_181024.2 | F: TGCTGTGGGGATGTCTCATA  R: GGTCAGCAGACTCTGGGTTC |
| *MLXIPL* | Carbohydrate-responsive element-binding protein Carbohydrate-responsive element-binding protein | NM 001205408.1 | F: ATCCGCCTCAACAACGC  R: TCCCTCCAAGACGACG |
| *ACOX1* | Acyl-CoA oxidase 1 | NC 015500.1 | F: TAAGCCTTTGCCAGGTATT  R: ATGGTCCCGTAGGTCAG |
| *ACSL* | Acyl-coA synthetase long chain Family Member 1 | NM 001076085.1 | F: CAAGCCTCCAGTACCTGAAGATC  R: AACGTGTTCTCTGTCATTTTCACAA |
| *FABP1* | Fatty acid binding protein 1 | NC 007309.4 | F: AAGTACCAAGTCCAGACCCAG  R: CACGATTTCCGACACCC |
| *CPT1A* | Carnitine palmitoyltransferase 1A | NC 007330.4 | F: GGTCAACAGCAACTACTACG  R: TGAACATCCTCTCCATCTGG |
| *CPT2* | Carnitine palmitoyltransferase 2 | NM 001045889.1 | F: ACGCCGTGAAGTATAACCCT  R: CCAAAAATCGCTTGTCCCTT |
| *TNF* | Tumor necrosis factor α | NM_173966.3 | F: CCATCAACAGCCCTCTGGTT  R:GGGCTACCGGCTTGTTACTT |
| *IL6* | Interleukin 6 | NM_173923.2 | F:GCCCAAGGTTAACGCTACAG  R:TGATCTCTCTGGGGTTCAGG |
| *NFKB1* | nuclear factor kappa B subunit 1 | NM_001076409.1 | F:CACGTATGGCGGAATTACCT  R:GCCACAACTTTCAGGGTCAT |
| *IL1B* | Interleukin 1 beta | NM_174093.1 | F:CCATGGAGAAGCTGAGGAAC  R:GGAGGACGTTTCGAAGATGA |
| *TLR4* | Tall like receptor 4 | NM_174198.6 | F:AGGCAGCATACTTCTCCA  R:GCCCTGAAATGTGTCGTCTT |
| *CD14* | CD14 molecule | NM_174008.1 | F:CCGACAACCAGAGAGAGG  R:CAGACACACGCAGCAGTGAC |
| *SAA2* | Serum amyloid A2 | NM_001075260.2 | F: CAGCCAGTGGATGTCCTTCT  R: GCACCCTTGTAGTTGGCTTC |
| *HP* | Haptoglobin | NM_001040470.2 | F: CCTATTACACACTGCGCACCT  R: GCACACTGCCTCACATTCAG |
| *PTGS2* | Prostaglandin-endoperoxide synthase 2 | NM_174445.2 | F: CCAGACAAGCAGGCTAATCC  R: GCAGCTCTGGGTCAAACTTC |
| *PLA2G2A* | Phospholipase A2 group IIA | NM_001075820.2 | F: AGATGAAGACCCTCCTGCTG  R: CTTTCCTGTCGTGTGCTTGA |
| *LIPE* | lipase E, hormone sensitive type | NM_001080220.1 | F: TCGTGGCTCAACTCCTTCTT  R: AGGGCTGCTTCAGACACACT |
| *FABP4* | fatty acid binding protein4 | NM_174313.2 | F: TTCAAGCTGGGAGTCGAGTT  R: TGTCCATTCCACTTCTGCAC |
| *FASN* | Fatty acid synthase | NM_001012669.1 | F: ACCTCGTGAAGGCTGTGACTCA  R: TGAGTCGAGGCCAAGGTCTGAA |
| *IL10* | Interleukin 10 | NM_174088.1 | F: CTGTATCCACTTGCCAACCA  R:AAGCTGTGCAGTTGGTCCTT |

Amplicon size was 90–120 bp in all primers. The primers were all used in a final concentration of 1 µmol/L. The thermal profile was composed of 15 min in 95 °C followed by 40 cycles of 15 s in 95 °C and 1 min in 60 °C

**Table S2**. List of immunoblot Ab's used to measure protein abundance

| Antigen | Abbreviation | Host | Dilution | Source |
| --- | --- | --- | --- | --- |
| Cannabinoid receptor1 | CB1 | Rabbit | 1:200 | Abcam biotech, Cambridge, UK, ab23703 |
| Cannabinoid receptor2 | CB2 | Rabbit | 1µg/ml | Enzo, NY, USA, ADI-905-820-100 |
| Monoglyceride lipase | MGLL | Rabbit | 1:200 | Abcam biotech, Cambridge, UK, ab24701 |
| Fatty acid amide hydrolase | FAAH | Rabbit | 1µg/ml | Aviva systems biology, CA, USA, ARP33121_P050 |
| Diacylglycerol lipase alpha | DAGLA | Goat | 0.3µg/ml | Aviva systems biology, CA, USA, OAEB01139 |
| Tumor necrosis factor α | TNF-α | Rabbit | 1:1000 | Aviva systems biology, CA, USA, OACA04183 |
| Nuclear factor kappa B | NFκB | Rabbit | 1:1000 | Cell signaling, MA, USA, 4717 |
| Interleukin 10 | IL-10 | Rabbit | 1µg/ml | Aviva systems biology, CA, USA, OACD04543 |
| Hormone sensitive lipase | HSL | Rabbit | 1:1000 | Cell signaling, MA, USA, 4107P |
| Fatty acid synthase | FASN | Rabbit | 1:2000 | Abcam biotech, Cambridge, UK, ab99359 |

**Table S3**. Fatty acid (FA) profile in plasma of postpartum dairy cows supplemented with n-3.

| **FA, %** | | **Treatment^1^** | |  | ***P*-value** |
| --- | --- | --- | --- | --- | --- |
|  | | **CTL** | **FLX** | **SEM** |  |
| C14:0 | 1.1 | 0.9 | 0.07 | 0.16 |  |
| C16:0 | 17.3 | 17.3 | 0.80 | 0.99 |  |
| C16:1 | 0.6 | 0.4 | 0.07 | 0.12 |  |
| C16:2 | 1.5 | 1.5 | 0.11 | 0.65 |  |
| C16:3 | 1.8 | 1.5 | 0.15 | 0.17 |  |
| C18:0 | 12.8 | 14.1 | 0.56 | 0.12 |  |
| C18:1n-9 | 8.6 | 6.9 | 1.05 | 0.27 |  |
| C18:1n-7 | 1.9 | 3.3 | 0.96 | 0.35 |  |
| C18:2n-6 | 44.0 | 41.5 | 2.13 | 0.44 |  |
| C18:3n-6 | 0.7 | 0.7 | 0.06 | 0.96 |  |
| C18:3n-3 | 1.8^a^ | 3.9^b^ | 0.15 | <0.0001 |  |
| C20:1n9 | 0.2 | 0.2 | 0.03 | 0.62 |  |
| C20:2n-6 | 1.1 | 1.0 | 0.10 | 0.51 |  |
| C20:3n-6 | 1.4 | 1.3 | 0.12 | 0.41 |  |
| C20:4n-6 | 2.0 | 2.1 | 0.14 | 0.85 |  |
| C20:4n-3 | 0.2 | 0.3 | 0.02 | 0.13 |  |
| C20:5n-3 | 0.3^a^ | 0.4^b^ | 0.03 | <0.001 |  |
| C22:5n-3 | 0.3 | 0.2 | 0.02 | 0.38 |  |
| C22:6n-6 | 0.2 | 0.3 | 0.05 | 0.30 |  |
| C22:6n-3 | 0.1 | 0.1 | 0.02 | 0.67 |  |
| C24:1n-9 | 2.2 | 2.1 | 0.40 | 0.97 |  |
| Saturated FA | 31.1 | 32.3 | 1.31 | 0.55 |  |
| MUFA^2^ | 13.5 | 12.9 | 0.74 | 0.62 |  |
| PUFA^3^ | 52.1 | 51.8 | 1.88 | 0.93 |  |
| n-3 | 2.6^a^ | 4.9^b^ | 0.17 | <0.0001 |  |
| n-6 | 49.4 | 46.9 | 1.96 | 0.38 |  |
| n-6:n-3 | 24.2 | 9.9 | 5.32 | 0.07 |  |

^1^Dairy cows were divided into two nutritional groups from –21 to 60 days PP; 1) Control group (CTL)­—a standard Israeli diet, 2) FLX—a standard diet supplemented with flaxseed oil containing n-3. *n*=8 per treatment ^2^monounsaturated FA; ^3^polyunsaturated FA

**Table S4**. White blood cells (WBC) average gene transcription levels (relative quantities; RQ) of ECS-related genes during the 1^st^ week PP of dairy cows supplemented with n-3 fatty acids.

| **Gene** | |  | | **Treatment^1^** | | | | **SEM** | | ***P*-value** | |
| --- | --- | --- | --- | --- | --- | --- | --- | --- | --- | --- | --- |
|  | | **Control** | | | **FLX** | |  | |  | | |
| *CNR1^2^* | | 0.01 | | | 0.00 | | 0.001 | | | 0.113 | |
| *CNR2^3^* | | 0.04 | | | 0.04 | | 0.012 | | | 0.637 | |
| *MGLL^4^* | | 0.03 | | | 0.01 | | 0.008 | | | 0.159 | |

^1^Dairy cows were divided into two nutritional regiment groups from –21 to 60 days PP; 1) Control group (CTL)—a standard Israeli diet, 2) FLX—a standard diet supplemented with flaxseed oil containing n-3. *n*=6 per treatment. ^2^Cannabinoid receptor 1; ^3^Cannabinoid receptor 2; ^4^Monoglyceride lipase

**Table S5**. Adipose tissue relative protein abundance of ECS-related, lipid metabolism and inflammatory proteins of PP dairy cows supplemented with n-3 fatty acids

|  | | |  | | **Treatment^1^** | | | | | | |  |  | | |  | | |
| --- | --- | --- | --- | --- | --- | --- | --- | --- | --- | --- | --- | --- | --- | --- | --- | --- | --- | --- |
|  | | **Control** | | | | **FLX** | | | **SEM** | | | | ***P*-value** | | | | |  |
| **Protein abundance, AU** | | |  | | | |  | |  | |  | | | |  | |  |  |
| CB1^2^ | | | 1.0 | | | | 0.8 | | 0.14 | | | | 0.37 | | | | | |
| DAGLA^3^ | | | 1.0 | | | | 1.5 | | 0.40 | | | | 0.41 | | | | | |
| FAAH^4^ | | | 0.7 | | | | 0.9 | | 0.22 | | | | 0.72 | | | | | |
| HSL^5^ | | | 1.4 | | | | 0.7 | | 0.31 | | | | 0.16 | | | | | |
| FASN^6^ | | | 1.5 | | | | 0.8 | | 0.40 | | | | 0.28 | | | | | |
| TNF-α^7^ | | | 1.1 | | | | 1.3 | | 0.26 | | | | 0.50 | | | | | |
| NFkB^8^ | | | 1.3 | | | | 1.8 | | 0.40 | | | | 0.37 | | | | | |
| IL-10^9^ | | | 1.0 | | | | 1.6 | | 0.27 | | | | 0.19 | | | | | |

^1^Dairy cows were divided into two nutritional regiment groups from –21 to 60 days PP; 1) Control group (CTL)—a standard Israeli diet, 2) FLX—a standard diet supplemented with flaxseed oil containing n-3. ^2^Cannabinoid receptor 1; ^3^Diacylglycerol lipase alpha; ^4^Fatty acid amide hydrolase; ^5^Hormone sensitive lipase; ^6^Fatty acid synthase; ^7^Tumor necrosis factor α; ^8^Nuclear factor kappa-light-chain-enhancer of activated B cells; ^9^Interleukin 10. Full blots are presented in supplementary Fig. S4

**Table S6**. Liver tissue relative protein abundance of ECS-related, lipid metabolism and inflammatory proteins of PP dairy cows supplemented with n-3 fatty acids

|  | | |  | | **Treatment^1^** | | | | | |  |  | | |  | | |
| --- | --- | --- | --- | --- | --- | --- | --- | --- | --- | --- | --- | --- | --- | --- | --- | --- | --- |
|  | | **Control** | | | | **FLX** | | **SEM** | | | | ***P*-value** | | | | |  |
| **Protein abundance, AU** | | |  | | | |  |  | |  | | | |  | |  |  |
| FAAH^2^ | | | 2.8 | | 2.1 | | | 0.58 | | | | 0.43 | | | | | |
| TNF-α^3^ | | | 1.2 | | 1.4 | | | 0.27 | | | | 0.67 | | | | | |
| IL-10^4^ | | | 3.3 | | 3.9 | | | 0.86 | | | | 0.68 | | | | | |

^1^Dairy cows were divided into two nutritional regiment groups from –21 to 60 days PP; 1) Control group (CTL)—a standard Israeli diet, 2) FLX—a standard diet supplemented with flaxseed oil containing n-3. ^2^Fatty acid amide hydrolase; ^3^Tumor necrosis factor α; ^4^Interleukin 10. Full blots are presented in supplementary Fig. S5
